# Editorial: Harnessing microbes for eco-friendly nanoparticle production and sustainable applications

**DOI:** 10.3389/fmicb.2025.1687584

**Published:** 2025-09-25

**Authors:** Divjot Kour, Amrik Singh Ahluwalia, Ajar Nath Yadav, Abdelhadi Abdallah Abdelhadi

**Affiliations:** ^1^University Centre for Research and Development, Chandigarh University, Gharuan, Mohali, Punjab, India; ^2^Department of Botany, Panjab University, Chandigarh, India; ^3^Department of Biotechnology, Dr. Khem Singh Gill Akal College of Agriculture, Eternal University, Baru Sahib, Himachal Pradesh, India; ^4^Department of Biotechnology, Graphic Era Deemed to be University, Dehradun, Uttarakhand, India; ^5^Faculty of Agriculture, Cairo University, Giza, Egypt

**Keywords:** biotechnological potential, commercialization, green synthesis, microbial nanotechnology, sustainable future

Industrialization and urbanization are major causes of environmental deterioration. There is a need for apt and sustainable alternatives to overcome environmental challenges. The emerging field of nanotechnology is transforming every aspect of human life. It is being recognized as the industrial revolution of the 21st century. It can transform the way society manufactures goods and provide better solutions to major environmental issues such as waste management and pollution. In recent years, nanostructured materials have attracted considerable interest due to their unique features compared to their polycrystalline counterparts (Khan et al., 2024). The ability to tailor the morphology, microstructure, composition, and physicochemical characteristics of nanomaterials through well-controlled approaches makes this field even more fascinating (Liu et al., 2016). Moreover, the growing need for sustainable, non-toxic and ecologically safe methods of nanoparticles (NPs) synthesis to reduce the negative environmental impacts while increasing energy productivity is a major area of research (Kour et al., 2024). High-energy physical and chemical procedures involving the use of toxic chemicals have been employed for the synthesis of NPs. These approaches result in high production costs and pose environmental risks. Green synthesis of NPs that exploits the metabolic potential of microbial entities such as actinomycetes, algae, bacteria and fungi is a promising approach for overcoming ecological challenges. Microbial communities are gifted with the innate ability to biosynthesize NPs and can be regarded as valuable biofactories for NP synthesis (Purohit et al., 2019).

This Research Topic was designed in Microbiotechnology to highlight the potential applications of the emerging area of research “Microbial Nanotechnology” for future sustainability. The articles in this Research Topic discuss microbe-mediated NP synthesis and their role as antimicrobial agents, in biofuel production and other applications. The original research by Sharmila et al. focused on green synthesis routes of antimicrobial compounds NPs from endophytes and antagonistic microbes as an innovative strategy for managing plant diseases caused by various phytopathogens including bacteria, fungi and viruses. The second research article by Do et al. explored the green synthesis of AgNPs using extracellular polymeric substances produced by *Graesiella emersonii* KNUA204. The findings of the study suggest the microalgal strain's potential for dual biomass utilization, integrating biofuel production with nanomaterial synthesis. Plokhovska et al. synthesized AgNPs from the plant growth-promoting bacterium *Pseudomonas* sp. Z9.3 and highlighted the potential of biosynthesized NPs as antimicrobial agents. Another original work by Gu et al., presented a novel concept of quantum dot synthesis mediated by *Lysinibacillus boronitolerans* QD4. Quantum dots are special nanomaterials that differ from bulk materials. They show unique optical and electronic properties due to quantum confinement, which confers them discrete energy levels. Microbe-mediated synthesis of quantum dots is an economical, environmentally-friendly production method with an extensive range of industrial applications. Rai et al., reviewed pycnidial fungi in the biosynthesis of NPs and highlighted their important applications in different sectors such as agriculture, the environment, industry, and medicine.

The utilization of microbes for the creation of NPs for promising applications, such as enhancing plant defenses against biotic stress, combating abiotic stress, nano-bioremediation and more is a new development in the realm of biotechnology and a breakthrough for advanced research in nanotechnology (Salem, 2023). The integration of diverse microbial groups and enzymes with nanotechnology provides a more sustainable method for the bioremediation of industrial effluents, and this approach can be scaled up for commercial use. It can even be extended to biohydrogen and bioelectricity generation from industrial waste, which would boost the industrial economy through green energy generation (Mandeep and Shukla, 2020). The integration of agri-nanotechnology generates a plethora of new possibilities to address global challenges in food production and sustainability (Mishra et al., 2017). NPs can be used in the synthesis of nanocapsulation and nanoformulations for next-generation pesticides and fertilizers which provide site-specific, controlled delivery of active ingredients to protect plants against drought, temperature fluctuations, and phytopathogens. NP-based smart delivery systems in the form of nanopesticides and nanofertilizers open new avenues for agro-sustainability (Kashyap et al., 2018).

The cellular, biochemical, and molecular mechanisms that mediate the biosynthesis of NPs should be studied to improve their synthesis rate and characteristics. Synthetic biology approaches and the engineering of microbial pathways to produce more valuable NPs equipped with novel functions for agriculture, the environment, industry, and scientific research will be useful in the future (Carmona et al., 2023). Taken together, the articles in this Research Topic highlight the potential of green nanotechnology for a sustainable future. Although nanotechnology provides innovative and promising solutions, it is important to understand the short and long-term impacts of NPs on humans and the environment to fully explore this technology's valuable impact on societal progress (Babatunde et al., 2020). Collaborative efforts between governmental regulatory agencies and the scientific community are fundamental to product design, development and commercialization, and acceptance by society.
